# Drug-Induced Acute Pancreatitis in Hospitalized COVID-19 Patients

**DOI:** 10.3390/diagnostics13081398

**Published:** 2023-04-12

**Authors:** Daniel Paramythiotis, Eleni Karlafti, Kalliopi Veroplidou, Maria Fafouti, Georgia Kaiafa, Smaro Netta, Antonios Michalopoulos, Christos Savopoulos

**Affiliations:** 1 First Propaedeutic Department of Surgery, AHEPA University General Hospital, Aristotle University of Thessaloniki, 54636 Thessaloniki, Greece; 2 Emergency Department, AHEPA University General Hospital, Aristotle University of Thessaloniki, 54636 Thessaloniki, Greece; 3 First Propaedeutic Department of Internal Medicine, AHEPA University General Hospital, Aristotle University of Thessaloniki, 54636 Thessaloniki, Greece

**Keywords:** drug-induced acute pancreatitis, COVID-19, SARS-CoV-2, pancreatic injury, pancreatic damage, COVID-19 treatment, COVID-19 drugs

## Abstract

Coronavirus disease-19 (COVID-19), caused by SARS-CoV-2, is a systemic disease that affects not only the respiratory system, but also other systems, including gastrointestinal. A great number of different drugs have been used on hospitalized patients for the management of COVID-19, and acute pancreatitis (AP) has been reported as a complication or side effect of these drugs. The development of drug-induced acute pancreatitis (DIAP) follows a complex of pathophysiological mechanisms, and particular risk factors play a key role. Diagnosis of DIAP depends on specific criteria, and based on these, a drug may be characterized as having a definite, probable or possible connection with AP. The aim of this review is to present the medications that are used for COVID-19 management and are associated with AP in hospitalized patients. The list of these drugs mainly includes corticosteroids, glucocorticoids, non-steroidal anti-inflammatory drugs (NSAIDs), antiviral agents, antibiotics, monoclonal antibodies, estrogens and anesthetic agents. Moreover, the prevention of the development of DIAP is vital, especially for critically ill patients who may receive multiple drugs. DIAP management is mainly non-invasive and the first step concerns the exception of the suspicious drug from patients therapy.

## 1. Introduction

Severe acute respiratory syndrome coronavirus 2 (SARS-CoV-2) is responsible for the coronavirus disease-19 (COVID-19) pandemic [1]. The clinical manifestations of COVID-19 vary from asymptomatic cases to severe respiratory infections [2]. Acute respiratory distress syndrome (ARDS) is the most common and life-threatening complication, while other complications include arterial and venous lung vasculopathy, thrombosis of smaller lung vessels, cardiac complications and renal and pancreatic injuries [3,4]. SARS-CoV-2 and certain drugs that have been administered to hospitalized COVID-19 patients were associated with the development of diseases and, more specifically, of acute pancreatitis (AP) [2].

AP is an inflammatory disease of the pancreas, without critical complications in 80% of patients [5]. In a minority of cases (20%), it may progressively lead to irreversible destruction of the pancreatic parenchyma and be life threatening [5,6]. The principle clinical symptom of AP is abdominal pain in a belt-like manner that is detected at the upper abdomen and radiates to the back [7]. Additional symptoms that often coexist are nausea, fever with a grade variation (from low to moderate) and vomiting [7,8]. AP can be developed due to several causes [8,9]. The most frequent of them are the blockage of the common bile duct by stones (40%) and alcohol abuse (35%) [7]. Medications have been recognized as an additional, but less common, cause of AP (2%) [7,8,9].

Depending on the Revised Atlanta Classification (RAC), for diagnosis of AP, two out of three of the following criteria are required to be present: (a) abdominal pain in accord with AP; (b) elevated pancreatic serum enzymes, with amylase or lipase three times above the normal rate; and (c) typical findings of AP using diagnostic imaging techniques, such as Computed Tomography (CT), Magnetic Resonance Imaging (MRI) scans or abdominal ultrasonography [8,10].

Moreover, SARS-CoV-2 is an RNA virus that attaches to the angiotensin-converting enzyme 2 (ACE2) receptor via a spike protein and invades the cells [3]. These proteins are mainly expressed in lung parenchyma [11], gastrointestinal luminal cells and pancreatic ductal, acinar and islet cells [1]. In addition to ACE2, transmembrane serine protease 2 (TMPRSS2) assists viral entry and is expressed in ductal cells [1]. One of the causes of AP might be the infection of those cells, though the exact mechanism of how SARS-CoV-2 infection affects pancreatic cells is not known yet [10]. According to some reports, the uncontrolled cytokine storm that develops due to SARS-CoV-2 infection could contribute to the severity of the AP [12]. Another hypothesis is that pancreatic ischemia may be the cause of different degrees of AP [10]. In addition, the inflammatory process that activates the coagulation cascade may potentially lead to an imbalanced hemostasis and increased coagulation. It is noteworthy that infection-related hyperglycemia has intense inflammation-promoting effects and, consequently, increases the quantity of inflammatory mediators. All the above mechanisms contribute to the manifestation of AP, each to a different degree [12].

**Figure 1:**
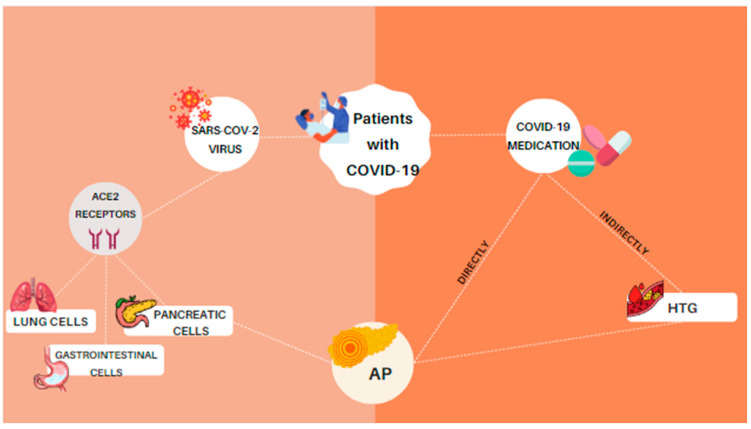
SARS-CoV-2 invades the host cells using ACE2 receptors, which are normally expressed not only in lung parenchyma, but also in pancreatic and gastrointestinal cells. A potential cause of AP is the infection of pancreatic cells, though another, less common cause is related to the medications that patients who are hospitalized with COVID-19 receive. These drugs may cause direct or indirect pancreatic damages via hypertriglyceridemia.

Furthermore, it has been found that COVID-19 patients who were diagnosed with pancreatic injuries also had elevated serum amylase or lipase [13,14]. Interestingly, apart from the direct viral damage in pancreatic cells and the thrombogenic state of COVID-19, the development of AP in COVID-19 patients may be related to the drugs they received during their hospitalization [1]. Drug-induced acute pancreatitis (DIAP) is not a common condition, representing less than 5% of all cases of AP, while at the same time, over 525 different drugs have been reported by World Health Organization (WHO) as suspected causative agents [15,16]. A great number of these drugs have been administered to COVID-19 patients and have led to direct or indirect pancreatic damage, mainly through the development of hypertriglyceridemia (HGT) [15,17], as is presented in Figure 1. Pathogenesis of DIAP involves certain mechanisms, such as direct toxic effects, accumulation of toxic metabolites or intermediates, immune response and hypersensitivity reaction [18]. Due to the infrequent presence of DIAP, there are not adequate scientific studies or accurate information, especially about newer drugs that have been suspected to lead to AP [19,20]. Therefore, information has been collected in case reports after the drugs have been used or by local drug safety committees [19,21].

The aim of this review is to summarize and analyze the drugs that have been used for the treatment of COVID-19 infections and are associated with DIAP in hospitalized COVID-19 patients.

## 2. Materials and Methods

Extensive research was performed on DIAP due to COVID-19 medications. The literature was searched up until 25 December, 2022, using the PubMed library and the following combination of the keywords: ‘acute pancreatitis COVID-19’ and ‘drug-induced pancreatitis’. In order to present a thorough depiction of DIAP in COVID-19 patients, no geographical restrictions were set, nor were limitations set for the number of papers that were included.

The aim of this review was to explore the drugs that contribute to the development of AP in hospitalized COVID-19 patients. 

### Results

**Figure 2:**
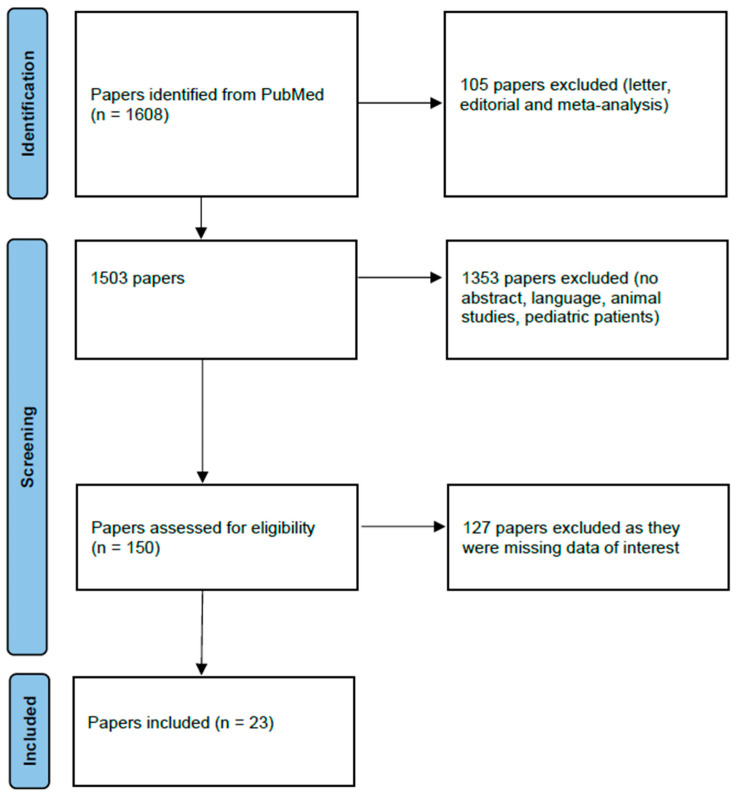
PRISMA 2020 flow diagram. This diagram illustrates the process followed for the collection and selection of papers used in our review.

A total of 1608 results were found after searching the keywords in the PubMed library. A total of 105 papers were excluded, as they concerned letters, editorials and meta-analyses. Another 1353 were excluded due to the absence of abstracts or due to the language (only papers written in English were included). In addition, animal studies or those that referred to pediatric patients were not included. Irrelevant papers were excluded as well. Full-text screening was performed, and 127 of the papers were excluded due to missing data of interest. Finally, 23 papers were included. The screening process of the papers selected for inclusion in this review is illustrated in the PRISMA 2020 flow chart (Figure 2).

## 3. Discussion

### 3.1. Diagnosis οf Drug-Induced Acute Pancreatitis

**Figure 3:**
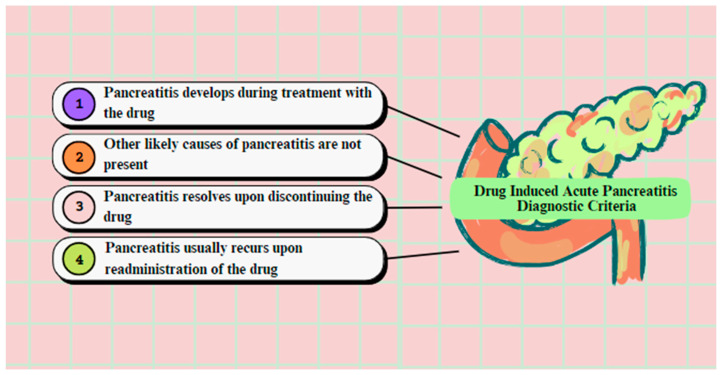
Diagnostic criteria of drug-induced acute pancreatitis.

The diagnostic criteria in order to prove that a specific drug induces AP, as suggested by Mallory and Kern, were: (a) pancreatitis should have developed during the treatment with the drug; (b) other probable etiologic agents of pancreatitis, such as gallstones or alcohol abuse, should not exist; (c) there was remission of pancreatitis after the termination of the drug administration; and (d) there was recurrence of pancreatitis with the re-administration of the drug (Figure 3) [19,21,22]. It is important to mention that an increase in levels of serum amylase and/or lipase is observed in COVID-19 positive patients and develops due to the pancreatic injury caused by the virus. Interestingly, this criterion is not diagnostic for AP in those patients, due to the fact that SARS-CoV-2 virus is also responsible for other gastrointestinal pathologies, such as gastroparesis, gastritis, enteritis and colitis, which may cause this rate elevation as well [23]. 

Based on the presence of these criteria, a drug could be characterized as having a definite, probable or possible connection with AP [21]. Thus, if all of the criteria are present, there is a definite association with AP. Likewise, a probable association is proved when some, but not all, of the above conditions do apply. Moreover, a drug is a potential cause of AP when the published evidence about the drug administration and the development of AP is incomplete and contradictory, because AP could have been triggered by the clinical condition of the patient or other types of therapy administered to them [19,22].

### 3.2. Risk Factors of Drug-Induced Acute Pancreatitis

**Figure 4:**
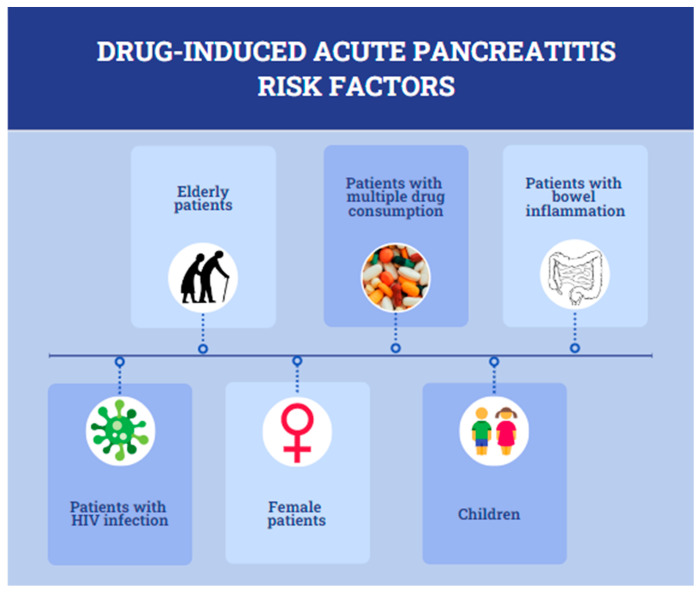
Drug-induced acute pancreatitis risk factors.

In consideration of epidemiological studies, there are several patient profiles that are expected to develop AP caused by drugs [19]. Based on Balani et al., elderly patients who receive multiple drugs are at a high risk of developing AP. In this case, the drugs for chronic diseases, coupled with the drugs for COVID-19 treatment during their hospitalization, could cause a severe pancreatic injury as a result of the patients’ metabolic reduction in drug elimination due to old age [9,18,24]. Furthermore, patients with bowel inflammation, individuals with advanced HIV infections and patients being treated with chemotherapeutic agents are considered to have high chances for drug-related AP [18,24]. In addition, children and females belong to the category of patients with a higher risk of developing DIAP [18,19,24]. The following figure (Figure 4) presents all of the above risk factors that contribute to the development of AP. Therefore, we strongly recommend the inclusion of COVID-19 infection in DIAP risk factors.

### 3.3. Drug Classification 

According to Badalov et al., there is an additional classification of four classes of drugs that can cause pancreatitis with regard to the quantity and other characteristics of evidence that have been reported [25]. 

Class I includes drugs with—at the minimum—one case report of AP, with a re-challenge of the drug and two subcategories. Class Ia consists of medications with at least one case report and a re-challenge, excluding all other etiologies of AP, such as gallstones, alcohol and hypertriglyceridemia. Class Ib includes medications with at least one case report and a confirmed re-challenge, but other causes are not excluded. Drugs with at minimum four cases and reported with a consistent latency period for at least 75% of the cases, are included in Class II. Class III consists of drugs with at least two case reports and neither a re-challenge nor a consistent latency among them. Finally, Class IV includes drugs with a single case report and no data concerning re-challenges [9,18,25].

### 3.4. Associated Medications

A variety of different drugs have been administered to COVID-19 patients, but some of them belong to the list of 525 drugs that have been reported by the WHO as suspected causative agents of AP [15]. These drugs are summarized in Table 1 and include antiviral agents such as lopinavir/ritonavir, remdesivir and interferons; anti-inflammatory drugs such as dexamethasone, glucocorticosteroids and non-steroidal anti-inflammatory drugs (NSAIDs); antibiotics such as azithromycin, ciprofloxacin and doxycycline; monoclonal antibodies such as tocilizumab; antipyretics such as acetaminophen; anesthetic agents such as propofol; and antihypertensive agents such as enalapril and lisinopril [3,25,26].

#### 3.4.1. Corticosteroids (Dexamethasone)

Corticosteroids are administered to patients for various conditions, such as rheumatoid arthritis (RA), asthma, septic shock, systemic lupus erythematosus, multiple sclerosis and lung tissue disorder, because of their immunosuppressive and anti-inflammatory properties [26,27]. Dexamethasone is an off-patent corticosteroid medication of low cost that was declared as a “major development” in the fight against SARS-CoV-2 [27].

The mechanism of action includes the blockade of two pathways of inflammation, vasodilation and immune cell migration. In particular, dexamethasone invades the cells, attaches to intracellular receptors and triggers an immune response. As a result, the drug suppresses the recruitment of pro-inflammatory cytokines, though it upregulates the gene expression of the immunomodulatory interleukin (IL)-10. Some of these cytokines, such as IL-1, IL-6 and TNF, have been associated with COVID-19-related mortality and greater severity of the disease [27,28]. 

The Randomised Evaluation of COVID-19 Therapy (RECOVERY) was a controlled, open-label trial that was conducted in the UK in order to estimate the effects of dexamethasone, among other drugs, on hospitalized COVID-19 patients. The trial included hospitalized COVID-19 patients from 176 National Health Service organizations of the UK and revealed that among 2104 patients who received 6 mg of dexamethasone once daily for 10 days, there was a lower mortality rate compared to the control group [29].

Nevertheless, corticosteroids seem to present various side effects, such as hyperglycemia and secondary infections [30]. In addition, dexamethasone has been associated with the development of AP [12,25]. According to Badalov et al., dexamethasone is classified as type Ib, as there was a case report of dexamethasone-induced AP with a positive rechallenge. However, other comorbidities, including alcohol abuse, were present [25].

In addition, there is a case of a 61-year-old man who was diagnosed with AP 14 days after his admission to the hospital due to COVID-19 [16]. His treatment included ciprofloxacin, acetaminophen, dexamethasone, pantoprazole and tocilizumab [13,16].

#### 3.4.2. Glucocorticosteroids (GCSs)

Glucocorticosteroids (GCSs) present anti-inflammatory and immunosuppressive properties and are used to treat various diseases, such as RA, asthma, multiple sclerosis and inflammatory bowel disease. Their mechanism of action includes interaction with the glucocorticoid receptors, which leads to the activation of immune cells, the inhibition of pro-inflammatory cytokines and chemokines, and the induction of proteins with anti-inflammatory activity [31]. Due to their properties, GCSs have been widely used for the treatment of SARS, Middle East Respiratory Syndrome (MERS) and COVID-19 [12,29]. National and international regulatory agencies suggest that GCSs should be used in SARS-CoV-2 therapy, along with oxygen therapy [31]. 

Long-term usage of GCSs may present various side effects that affect the cardiovascular, gastrointestinal, nervous, endocrine and immune systems [31]. However, some studies have shown that GCSs increase the risk of AP [12,32]. For that reason, it is necessary to monitor patients regularly for the development of AP. The fact that GCSs were used for severe cases of COVID-19 makes it difficult to determine the actual cause of AP, which could be a severe COVID-19 infection, GCSs administration or both of them [12].

#### 3.4.3. Non-Steroidal Anti-Inflammatory Drugs (NSAIDs)

**Figure 5:**
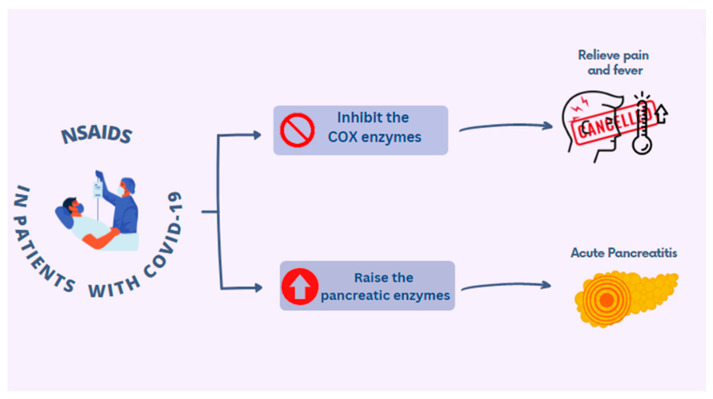
Non-steroidal anti-inflammatory drugs (NSAIDs) used for COVID-19 patients.

Non-steroidal anti-inflammatory drugs (NSAIDs) are widely used to relieve pain and reduce inflammation, mainly in chronic inflammatory syndromes, such as RA and osteoarthritis. They act by inhibiting the cyclooxygenase (COX) enzyme that is required to convert arachidonic acid into thromboxanes, prostaglandins and prostacyclins. Two different categories, COX-1 and COX-2 enzymes, are expressed as a result of different responses and involvement in different procedures. NSAIDS are characterized, as for the inhibition of COX, as either non-selective when they restrain both COX-1 and COX-2 or as COX-2-selective when they restrain the COX-2 type enzymes. Despite the fact that NSAIDs constitute one of the most commonly used drug categories, there is a risk of gastrointestinal complications, as well as cardiovascular and renal adverse effects with their chronic use [33]. Furthermore, due to their mode of operation [15,33], NSAIDs have been used on hospitalized patients during SARS-CoV-2 infections in order to relieve pain (especially headache) and fever, as is shown in Figure 5.

A threat of developing AP has been reported in relation to the administration of NSAIDs to COVID-19-positive patients [15,34]. In several reported cases of AP in patients with a SARS-CoV-2 infection, the consumption of NSAIDs was considered to be the main cause of the elevation of pancreatic enzymes, rather than the viral effects [35] (Figure 5). Based on Pezzilli et al., naproxen is recommended as analgesic agent because the danger of it leading to AP is lower [34].

#### 3.4.4. Antiviral Agents

Antiviral drugs are protease inhibitors, which inactivate the proteases and, thus, prevent the replication of the virus. They are used in the treatment of HIV, and it has been demonstrated that they can also prevent the viral replication of SARS-CoV-2, a procedure that is vital for the survival of affected patients [3]. This drug category contains lopinavir/ritonavir and remdesivir (antiviral nucleoside analog) drugs that are used in different stages of the COVID-19 disease, from the early stages with less severe symptoms to intense conditions, both at home and in the hospital [3,26]. However, as a side effect, they are suspected of causing AP in hospitalized patients with a SARS-CoV-2 infection, [2,23].

##### Lopinavir/Ritonavir

Lopinavir/ritonavir is an antiretroviral drug that acts as a protease inhibitor and has been used for the treatment and prevention of HIV [36,37]. The inhibition of the 3C-like proteinase (3CL^pro^) of coronavirus is the main reason that it is used for the treatment of COVID-19 [12,36]. Lopinavir/ritonavir has been related with a faster drop of fevers and a decrease in the viral load [37]. Despite the improvement of lung function, it cannot achieve the inhibition of viral replication. For that reason, since the disease may become severe, physicians need to take into consideration not only the benefits of treatment, but also the risks [23,36]. In addition, lopinavir/ritonavir has been associated with abnormalities in lipid metabolism; multiple gastrointestinal symptoms, such as diarrhea, abnormal stools, abdominal pain, nausea and vomiting [36,38]; hyperbilirubinemia and increased GGT (Gamma-Glutamyl Transpeptidase) [30].

Furthermore, according to Badalov et al., lopinavir/ritonavir is classified as a type IV drug, or one of the medications with the weakest associations to AP due to a lack of information [12,25]. A report by Rubel et al. described that 2 out of 47 COVID-19 patients with moderate/severe symptoms who were treated with lopinavir/ritonavir developed significantly high levels of triglycerides within 2 weeks of the treatment [38]. Hypertriglyceridemia is an established etiological factor in the pathogenesis of AP [17]. 

Moreover, a case report of a previously healthy 55-year-old COVID-19 patient showed that after five days of treatment with lopinavir/ritonavir, azithromycin and propofol, he demonstrated high levels of pancreatic enzymes. Ultimately, CT findings led to the diagnosis of AP [39].

##### Remdesivir

Remdesivir is a small-molecule monophosphoramidate pro-drug, which belongs to the nucleotide analogues class and blocks the RNA-dependent RNA-polymerase (RdRp) [3,26]. This molecule acts after the virus enters the host cell. After remdesivir enters the cells, it is cleaved to the nucleoside monophosphate analog and then phosphorylated again to result in its active triphosphate form (RDV-TP), which is similar to adenosine triphosphate (ATP). Competing with ATP, RDV-TP incorporates the RdRp and viral RNA chain complex, conducing to the untimely termination of the RNA transcription of the virus and RNA synthesis inhibition. Initially, remdesivir was synthesized to confront Ebola virus infections. Later, based on additional studies, it was proved to be suitable for coronavirus treatments, too, because of the fact that this medication could restrain the replication of the virus and lessen SARS-CoV-2-related lung disorders. Additionally, it has been reported that remdesivir is not recommended for the early stages of COVID-19 infection, but, due to the fact that it improves their condition in a short period of time and lessens the threat of the disease’s progress, it is necessary for patients with severe symptoms who require respiratory support [26].

According to Allam et al., when remdesivir is used in COVID-19 treatment, it is possible for AP to be induced through the elevation of serum triglycerides [2]. Furthermore, Miyazaki at al. revealed that remdesivir consumption during COVID-19 treatment raises the chances of developing AP or elevating the rate of the pancreatic enzymes [2,20].

#### 3.4.5. Tocilizumab

Tocilizumab (TCZ) is a humanized monoclonal antibody that competitively inhibits the binding of interleukin-6 (IL-6) to its receptor (IL-6R) and was approved in 2009 by the European Medicines Agency (EMA) for the treatment of RA [40]. TCZ is an antagonist of the IL-6 receptor, disrupts cellular signal transduction pathway and manages to decrease inflammatory responses [26,30]. Overexpression of IL-6 has pathological effects on chronic inflammation and autoimmunity [26]. The cytokine storm that develops during severe COVID-19 infections causes elevated levels of IL-6, C-reactive protein, D-dimers and ferritin [30]. During the COVID-19 pandemic, TCZ was the most-used drug for COVID-19 management, and studies have indicated that it contributed to diminish the need for intubation [3,30]. 

Even though it is a well-tolerated drug [40], a great number of adverse effects have been noted, including infections of the upper respiratory system, hypercholesterolemia, hypertriglyceridemia, arterial hypertension, gastrointestinal symptoms, stomatitis, thrombocytopenia, neutropenia, increased liver enzymes, rash, dizziness and headaches. Additionally, TCZ has been associated with AP [26,40]. According to Morrison et al., TCZ was received in combination with lopinavir/ritonavir by two critically ill COVID-19 patients treated in the intensive care unit (ICU). Three days after the initiation of TCZ significant increases were observed in their triglycerides, amylase and lipase. According to the Adverse Drug Reaction (ADR) Probability Scale by Naranjo, there was a probable relation between TCZ and hypertriglyceridemia in both patients. For that reason, COVID-19 patients who receive TCZ should be regularly monitored for hypertriglyceridemia [41]. 

#### 3.4.6. Antibiotics 

Antibiotics are drugs used for bacterial infections and act by killing bacteria or preventing their reproduction and growth. Traditionally, they were used to treat only certain infections caused by bacteria. Nevertheless, recent studies have shown that certain antibiotics slow the multiplication of some viruses, such as SARS-CoV-2 [42]. However, antibiotics may also be involved in the pathogenesis of AP, when they induce the elevation of serum triglycerides [15].

##### Azithromycin

Azithromycin is a macrolide that acts against a range of Gram-positive and Gram-negative bacteria [26], is broadly available and has a widely tested safe profile [42]. Azithromycin was found to regulate interleukin production and, as a result, helps to control the immune response and prevent COVID-19 symptoms [43]. In addition, it regulates the pH of endosomes and the trans-Golgi network in respiratory epithelial cells, which are a major target of SARS-CoV-2 [43,44]. Laboratory studies have found that azithromycin reduces respiratory viral activity and inflammation, and for that reason, it has been administered to COVID-19 patients [42,43]. Azithromycin, in combination with hydroxychloroquinine, has been used as a treatment for hospitalized COVID-19 patients [26]. A possible side effect of azithromycin, mainly when in combination with hydroxychloroquinine, is QT-prolongation causing cardiac arrhythmias and hepatotoxicity [26,30,42]. However, according to available data, the risk of azithromycin-induced AP is low [12].

With regards to azithromycin-induced AP, there are only few reports connected to it, and there is no direct data [12]. Interestingly, there was a case of a young girl with concomitant symptoms of COVID-19 and AP who received azithromycin, remdesivir and dexamethasone and eventually was treated for both conditions [45].

##### Ciprofloxacin

Ciprofloxacin is a broad spectrum antibiotic that belongs to fluoroquinolones which are used for the treatment of respiratory and urinary infections. Additionally, this type of drugs is used to treat hospital-acquired infections, when macrolides and β-lactam antibiotics are not effective due to antimicrobial resistance [46]. Ciprofloxacin is an appropriate option for patients with more than one infection or patients with predisposing factors for Gram-negative infections [47]. Its mechanism of action includes the inhibition of DNA replication and transcription [46,47]. 

It was found that ciprofloxacin attaches to the main protease (M^pro^) of SARS-CoV-2 and, consequently, it may prevent the viral replication. Ciprofloxacin, along with other respiratory fluoroquinolones, is characterized by an excellent safety profile and, thus, has been used to treat COVID-19 patients [48]. 

However, some serious adverse effects have been recognized, such as a prolonged QT interval, hyper or hypoglycemia and photosensitivity [47]. Furthermore, ciprofloxacin has been associated with the development of AP. There was a case of a healthy 61-year-old man who was diagnosed with AP fourteen days after his admission to the hospital due to a COVID-19 infection. The treatment included ciprofloxacin, acetaminophen, dexamethasone, pantoprazole and tocilizumab [16]. 

##### Doxycycline

Doxycycline is a well-tolerated broad-spectrum antibacterial agent that is classified to tetracyclines that present bacteriostatic properties and inhibit bacterial protein synthesis. Tetracyclines are used against Gram-positive bacteria, Gram-negative bacteria, intracellular organisms and protozoan parasites [42]. Doxycycline presents anti-inflammatory and antiviral properties against several RNA viruses. In particular, it may inhibit the viral papain-like protease (PL^pro^) and 3CL^pro^, which are necessary for the replication of SARS-CoV-2, and, thus, it has been used to treat COVID-19 patients [49,50]. Its mechanism of action involves the inhibition of metalloproteinases (MMP)-9 and IL-6, which both contribute to the blockade of the cytokine storms that arise due to severe COVID-19 infections [12,50]. However, there is not an established link between the administration of doxycycline and the amelioration of the severity of COVID-19 symptoms. [23]. 

In addition, there is a potential association between doxycycline and the development of AP [23,51]. According to a reported case, a 52-year-old woman who was hospitalized due to COVID-19 pneumonia developed AP after 15 days. The medications the patient received included doxycycline, dexamethasone, azithromycin and pantoprazole. All of these drugs have been linked to AP, and for that reason, it was considered a DIAP after other common causes of AP were ruled out [10].

#### 3.4.7. Propofol

Propofol is a sedative-hypnotic agent, commonly formulated in a 10% lipid emulsion, which is administrated intravenously (IV) and has been recommended by the Society of Critical Care Medicine’s 2018 clinical practice guideline for administration to patients who are in critical condition, require mechanical ventilation and are hospitalized in ICU [52].

Based on Kermad et al., COVID-19 positive patients who are in critical condition related to acute respiratory failure are in need of invasive respiratory support and adequate sedation to ensure essential gas exchange [15,53]. For that reason, propofol is used on patients who are in critical condition with a SARS-CoV-2 infection, and it has a major role in their therapeutic outcome [54]. However, it has been reported to cause hypertriglyceridemia when it is used at elevated infusion rates and for considerable periods of time on ICU patients [17,52].

According to Badalov’s classification of drugs that can cause AP, propofol belongs to Class II [25]. A considerable number of AP cases have been reported after the administration of this drug to ICU patients, and some of them were induced by the increase in triglyceride levels in the patients’ serums [23,55].

#### 3.4.8. Estrogens

Estrogens, a category of sex hormones, can be used as a hormone replacement in medical treatments to manage and cure menopausal symptoms. They can moderate the responses of the innate immunity, inflammation system and cytokine storms, as well as provoking the responses of B-cells and the production of antibodies [56]. Due to their action, estrogens have a strong effect in the medical care of COVID-19 patients, at both early and later stages of the viral infection, and also on the ARDS or multi-organ failures that might occur. Consequently, estrogens benefit patients and reduce mortality by improving their clinical conditions [56,57]. 

Despite the beneficial action of estrogens, there have been some case reports that demonstrate that estrogen treatments might lead to the pathogenesis of AP, and for that reason, this category of drugs belongs to Class Ib of the Badalov classification [17,25]. AP has been the result of the hypertriglyceridemic effects of the estrogens used for COVID-19 infections, as patients’ serum triglyceride levels were found to be elevated, and, likewise, familial hyperlipoproteinemia was found in them [17,25,55]. Interestingly, there are reported cases that the AP was not hypertrigliceridemia-associated, but rather induced by the estrogen [24,25].

#### 3.4.9. Other Medications

There are a number of other drugs that have been administrated to patients who are hospitalized with SARS-CoV-2 infections and that have been related to the development of AP, but there is not adequate knowledge about their involvement and not enough case reports to prove it [12,16].

Interferons (IFNs) are fundamental agents of the innate immune system, thanks to their action against viruses and their immunomodulatory functions [58]. IFN-β, used as a treatment for hospitalized patients who are COVID-19 positive, has been demonstrated to have significant efficacy, as it may diminish the hospitalization time, the ICU admission rate, the need for invasive respiratory support and the mortality rate of patients with severe COVID-19 [58,59]. Despite this, there are a few individual cases that report the onset of AP due to IFN, and for that reason, it is included in Class III of the Badalov classification of drugs [12,25].

Acetaminophen, used as non-etiotropic agent for the reduction of pain and fever and the control of the inflammatory response, has been proposed for the domiciliary management of pain and fever in the earlier stages of COVID-19 [60]. Nevertheless, in some cases in which this drug has been administrated to patients who displayed more serious conditions related to COVID-19, it has been associated with AP [13,16]. Badalov classified acetaminophen as Class II [25].

Additionally, there are several drugs either recently used and suggested for COVID-19 treatment or included in the chronic medication of some of the patients whose cases have been reported, such as lisinopril, enalapril, pantoprazole, asparaginase, which are connected to the development of AP. Moreover, it is possible that AP occurred when these drugs were used at the same time or in combination with some of the aforementioned drugs [16,17,23].

### 3.5. Prophylaxis

It is worth mentioning that, although drugs rarely constitute the etiological agent of the reported cases of AP, there are reports of mortalities from DIAP [18,24].

Moreover, as long as the treatment of the patient includes drugs that have strong evidence of causing AP, regular monitoring is recommended in case of the development of symptoms of abdominal pain and changes in serum amylase and lipase [18]. In addition, in order to prevent this adverse reaction, it is important to know the ways that the drugs induce pancreatitis [24]. Thus, for drugs that cause hypertriglyceridemia, such as lopinavir/ritonavir [23], remdesivir [2], tocilizumab [52], estrogens [55] and propofol [52], it is recommended that the serum triglyceride levels of every COVID-19-positive patient should be measured before the start of the therapy [2,24]. Furthermore, Allam et al. suggest that patients with hypertriglyceridemia should not use remdesivir treatment [2]. According to Kener et al., there are alternative sedative agents, such as dexmedetomidine, midazolam or lorazepam, that can be used instead of propofol for hospitalized patients with COVID-19 infections, depending on each patient’s condition and needs [52].

Hypersensitivity reactions, vascular thrombosis, constriction of the pancreatic duct, direct cellular toxicity, suppression of the immune system, osmotic or metabolic effects, accumulation of toxic metabolites and hepatic involvement are other mechanisms which are related to the induction of AP. Additionally, in some cases, there are idiosyncratic reactions that do not follow familiar action patterns; are not predictable and, so, cannot be prevented; and do not depend on the dose of the drug or occur only to susceptible patients [24].

Finally, when drugs are used on patients who are in danger of developing AP, it is a matter of great importance to consider the advantages and their disadvantages and choose the most proper treatment for each patient [18]. The re-challenge of a drug that is suspected to be associated with AP should only be ethically excused if there are no other appropriate medications that can displace it and after a benefit-risk analysis of the drug and the patient’s medical condition [9,24].

### 3.6. Therapy

Regardless of its etiology, AP management is mainly conservative, as long there are no further complications. Methods of management include fluid resuscitation, nutritional support, bowel rest, analgesia, antibiotics and avoidance of medications that could lead to pancreatic damage [8,15,23]. There is growing evidence that the severity of AP may depend on the uncontrolled cytokine storm that occurs due to COVID-19 infections, pancreatic ischemia and hemostasis imbalances. Current studies have shown that the suppression of coagulation may diminish the development of AP or even contribute to the treatment of the disease [10,12,61]. 

Clinical guidelines suggest that intravenous volume resuscitation should be initiated immediately after the diagnosis of AP. A low intravascular volume related to pancreatic, peripancreatic or systemic edema in patients with AP, or due to vomiting and reduced oral intake, increases the risk of complications and mortality [8]. Particularly, most guidelines recommend lactated ringer solution because of its anti-inflammatory properties and the reduced odds of patients developing systemic inflammatory response syndrome (SIRS), as opposed to normal saline [8,62,63]. In case of pre-existing kidney disease or heart failure, the risks of fluid overload should be considered [8]. It is crucial that the suspension of any medications already known to induce AP should be considered, taking into consideration the indications of each medication, the patient’s clinical status and the risk-benefit relationship between the two [23,24]. Pancreatitis could probably be considered to be drug-induced if symptoms cease after the discontinuation of the suspicious drug [19]. Nutritional support via oral or enteric routes should initiate as soon as possible for patients with AP [63], so as to counteract their catabolic states and reduce the chances of infectious complications. Specifically, enteral nutrition restores the loss of calories that occurs in cases of severe AP, increases blood flow to prevent damage to the intestinal mucosa and stimulates intestinal motility [8]. Patients with indications of organ dysfunction, mental status changes and renal insufficiency should be transferred to the ICU [24].

## 4. Conclusions

DIAP in hospitalized patients is not common, but its timely diagnosis is vital for each patient. The number of reported cases of DIAP in patients who are hospitalized with COVID-19 is small. Nevertheless, many drugs that are used for COVID-19 treatments for hospitalized patients are related to definite, probable or possible connections with AP. The administration of drugs that are associated with AP to patients who are hospitalized with COVID-19 is accompanied by a strong recommendation to closely monitor each patient in order to avoid potential complications that may lead to the irreversible destruction of the pancreas.

## Figures and Tables

**Table 1:** Drugs used for COVID-19 infections that may also associate with DIAP.

Medication	Type	Badalov Classification	References
Dexamethasone	Corticosteroids	Ib	[25,26]
Glucocorticosteroids	Corticosteroids	N/A	[26]
Non-steroidal anti-inflammatory drugs (NSAIDs)	Non-steroidal anti-inflammatory drugs (NSAIDs)	N/A	[15]
Lopinavir/ritonavir	Antiviral drug	IV	[3,12]
Remdesivir	Antiviral drug	N/A	[3,26]
Tocilizumab	Monoclonal antibody	N/A	[3]
Azithromycin	Antibiotic	N/A	[26]
Ciprofloxacin	Antibiotic	III	[16]
Doxycycline	Antibiotic	III	[10,16]
Propofol	Anesthetic agent	II	[25]
Estrogens	Hormones	Ib	[25]
Interferons	Antiviral agent	III	[25,26]
Acetaminophen	Antipyretic	II	[25]
Enalapril	Antihypertensive agent	Ia	[25]
Lisinopril	Antihypertensive agent	III	[25]
Asparginase	Enzyme	II	[25]

## Data Availability

Not applicable.
